# The Role of Gut Microbiota in Gastrointestinal Immune Homeostasis and Inflammation: Implications for Inflammatory Bowel Disease

**DOI:** 10.3390/biomedicines13081807

**Published:** 2025-07-24

**Authors:** Elisabetta Bretto, Miquel Urpì-Ferreruela, Gherzon Rimer Casanova, Begoña González-Suárez

**Affiliations:** Endoscopy Unit, Gastroenterology Department, Hospital Clínic of Barcelona, 08036 Barcelona, Spain; murpi@clinic.cat (M.U.-F.); gscasanova@clinic.cat (G.R.C.); bgonzals@clinic.cat (B.G.-S.)

**Keywords:** IBD, Crohn’s disease, ulcerative colitis, immunological modulation, microbiota, microbiome, inflammation, pathogenesis, flare immunity

## Abstract

Inflammatory bowel disease (IBD), a heterogeneous group of recurring inflammatory conditions of the digestive system that encompass both ulcerative colitis (UC) and Crohn’s disease (CD), pose a significant public health challenge, currently lacking a definitive cure. The specific etiopathogenesis of IBD is not yet fully understood, but a multifactorial interplay of genetic and environmental factors is suspected. A growing body of evidence supports the involvement of intestinal dysbiosis in the development of IBD, including the effects of dysbiosis on the integrity of the intestinal epithelial barrier, modulation of the host immune system, alterations in the enteric nervous system, and the perpetuation of chronic inflammation. A comprehensive understanding of these mechanisms is important to define preventive measures, to develop new effective and lasting treatments, and to improve disease outcome. This review examines the complex tri-directional relationship between gut microbiota, mucosal immune system, and intestinal epithelium in IBD. In addition, nonpharmacological and behavioral strategies aimed at restoring a proper microbial–immune relationship will be suggested.

## 1. Introduction

Inflammatory bowel diseases (IBDs) are chronic and recurring inflammatory conditions, encompassing Crohn’s disease (CD) and ulcerative colitis (UC) [1,2]. While both share similar clinical symptoms, CD starts as a disease entity limited to the ileocecum and can then affect any part of the gastrointestinal tract, whereas UC is limited to the colon and rectum [1,2]. They are stratified into mild, moderate, and severe categories based on clinical and endoscopic evaluations [3]. These diseases are marked by persistent inflammation, leading to complications such as hospitalization, surgery, and disability, significantly impacting the quality of life of affected individuals [4,5]. Furthermore, colitis promotes carcinogenesis by fostering the expansion of genotoxic bacteria, with UC patients facing a 20% risk of colorectal cancer (CRC) development and CD patients a 1.2% risk compared to general population [6]. Timely and effective treatment is crucial to prevent these complications and improve the well-being of IBD patients [7].

Globally, approximately 6.8 million individuals suffer from IBD, with an increasing incidence in developing nations and a stable trend in highly prevalent developed countries, imposing significant strain on healthcare resources [8,9]. Although the etiology of IBD remains elusive, emerging evidence suggests that genetic susceptibility, defects in mucosal barrier function, immune stimulation, and alterations in microbial composition and function of the intestinal environment contribute to its pathogenesis [10]. This is supported by the identification of over 200 IBD-associated susceptibility genes involved in host–microbiota interactions [11]. Consequently, the gut microbiota has emerged to play an important role in IBD, attracting considerable attention in research on IBD pathogenesis and biological therapies [12].

This review aims to synthesize current knowledge on the complex interplay between the gut microbiota and the initiation and progression of IBD, highlighting implications for diagnosis, treatment, and prevention. It offers novel insights by integrating recent advances into the immunomodulatory roles of bacterial, viral, fungal, and archaeal components of the gut microbiota, with particular emphasis on microbiota-derived metabolites and next-generation therapeutic strategies.

## 2. Immune Response and Disease Induction

The complex interaction among immune cells, cytokines, and molecular pathways lies at the heart of immune dysregulation in IBD, characterized by excessive activation of pro-inflammatory pathways and inadequate regulation of anti-inflammatory mechanisms [12,13]. Various immune cell types and signaling molecules contribute to this imbalance [12]. In CD heightened production of pro-inflammatory cytokines like tumor necrosis factor alpha (TNF-α), interleukin (IL)-1β, and IL-6 sustains chronic inflammation, while in UC abnormal activation of immune cells including T-cells, macrophages, and dendritic cells (DCs) leads to persistent mucosal inflammation [14,15].

Innate immune dysfunction plays a significant role, with DCs, macrophages, and epithelial cells detecting and responding to microbial components through pattern recognition receptors (PRRs) such as Toll-like receptors (TLRs) and nucleotide-binding oligomerization domain (NOD)-like receptors (NLRs) [16]. Dysregulation of these innate immune pathways can trigger an exaggerated immune response, contributing to chronic intestinal inflammation [16]. Genetic studies have identified susceptibility loci associated with CD, including NOD2/caspase recruitment domains (CARD) 15, autophagy-related (ATG) 16L1, and IL-23R, while genome-wide association studies in UC have identified genes like IL-23R, IL-10, and human leukocyte antigen (HLA) genes [17,18,19]. These genetic variants influence innate immune responses, autophagy, and the balance of pro- and anti-inflammatory cytokines [13,17,18,19].

Intestinal infections may also contribute to perpetuating inflammation [20]. Episodes of *Salmonella/Campylobacter* gastroenteritis have been linked to an increased risk of developing IBD, with alterations in the TLR4 gene potentially predisposing individuals to infections by these Gram-negative bacteria and increasing susceptibility to enteric infections in general [21,22]. Consequently, pathogenic infections may alter the composition of the commensal gut microbiota and disrupt commensal tolerance, leading to the chronic inflammation associated with IBD [23].

Furthermore, environmental factors such as smoking, diet, and the gut microbiota significantly contribute to IBD pathogenesis and progression through epigenetic modifications like DNA methylation, histone modifications, and the regulation of noncoding RNA [24]. These modifications alter gene expression patterns and immune responses in the intestinal mucosa, affecting epithelial barrier function and inflammatory pathways [24].

Lastly, bacterial metabolites may directly influence pro- and anti-inflammatory pathways, further contributing to the complex pathogenesis of IBD [25].

## 3. The Role of Gut Microbiota in IBD

The human gastrointestinal tract hosts a diverse ecosystem of microorganisms collectively known as the gut microbiota, comprising approximately 40 trillion microorganisms and containing about 150 times more genes than the human genome [26]. The gut microbiota consists of beneficial bacteria that contribute to gut mucosal homeostasis, as well as harmful bacteria, known as “pathobionts”, which can trigger gut inflammation and mucosal damage [26]. As a crucial component of the intestinal barrier, the gut microbiota has evolved alongside the host’s intestinal environment, contributing to the maintenance of epithelial mucosal homeostasis, immune regulation, metabolic balance, and nutrient provision in a healthy state [27]. It also plays a role in sustaining intestinal structure and defending against opportunistic pathogen invasion [27]. However, dysregulation of these interactions can lead to inflammation-related diseases [28].

The composition and function of the gut microbiota can be influenced by various factors such as diet, drug treatment, smoking, age, and genetics [29,30]. These factors may alter the symbiotic interplay between the microbiota and the host, contributing to the pathogenesis of inflammation-related diseases like IBD [31]. Disturbances in the balance between beneficial and harmful bacteria, as well as reductions in biodiversity and species richness within the microbial community, can disrupt physiological gut homeostasis and damage the intestinal mucosal barrier [32].

The integrity of the intestinal barrier is maintained through the interactions of various components, including the mucus layer, immunoglobulin A (IgA), antimicrobial peptides (AMPs), and intercellular tight junctions (TJs) [33]. The mucus layer serves to stabilize the intestinal lining, while AMPs, produced by intestinal epithelial cells (IECs), regulate microbial colonization in the gut lumen and prevent the infiltration of epithelial cells [33]. Intercellular TJs, comprising proteins located near the apical membrane of epithelial cells, play a crucial role in determining the physical integrity of the intestinal barrier [33]. Claudin proteins, integral components of TJs, serve as essential defenses against pathogen invasion [34]. Abnormal expression of claudin can result in reduced cell adhesion, structural damage, and impaired function of both epithelial and endothelial cells [35].

**Figure 1:**
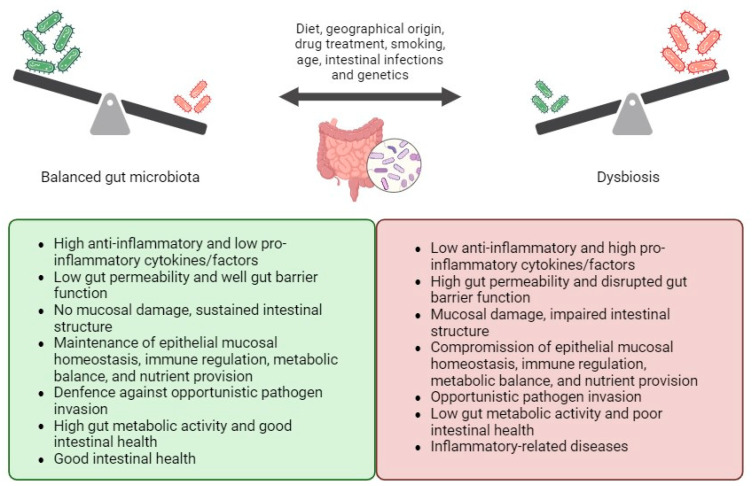
The gut microbiota of a healthy individual (**left**) compared to that of a patient with inflammatory bowel disease (IBD) (**right**).

Disruption of these interactions, commonly referred to as “leaky gut,” can exacerbate intestinal inflammation by allowing bacteria to translocate into the lamina propria, triggering an inflammatory response mediated by TLRs and NF-κB pathways [36]. This leads to the proliferation of pro-inflammatory T-cell subsets, including T helper Th1, Th2, and Th17 cells, which produce various pro-inflammatory cytokines, chemokines, and other mediators [36,37]. These molecules recruit pro-inflammatory cells to the gut mucosa, contributing to mucosal damage, while concurrently downregulating anti-inflammatory mediators such as T regulatory cells (Tregs) [32]. In this altered gut mucosa environment, “pathobionts” thrive and further enhance the immune response [38] (Figure 1).

**Figure 2:**
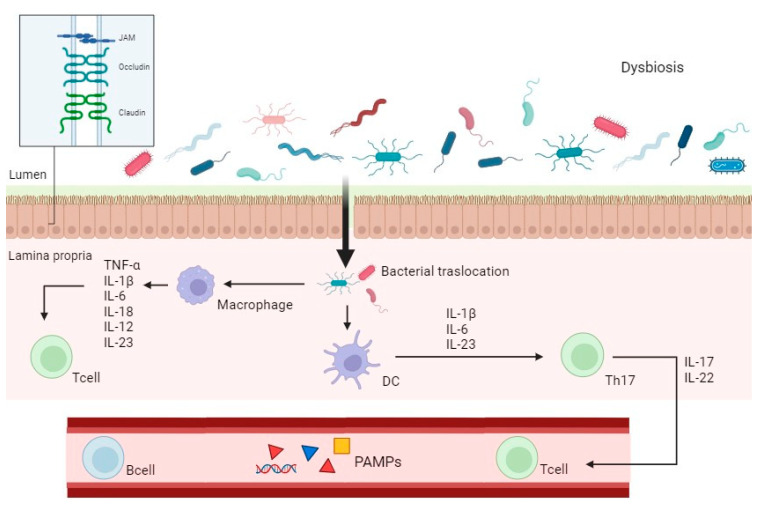
Dysbiosis, leaky gut, and pro-inflammatory intestinal and systemic response.

This complex pathological mechanism involving the immune system and gut microbiota is driven by the activation of specific receptors on immune cells, such as PRRs, which recognize pathogen-associated molecular patterns (PAMPs) and danger-associated molecular patterns (DAMPs) [39,40]. These receptors initiate innate immune responses against infectious agents [39,40] (Figure 2).

Comparing microbiota across individuals is challenging due to this complexity [41]. Additionally, while various molecular techniques have been employed to study the human gut microbiota, none can capture the full spectrum of microorganisms inhabiting the gut [42]. Nevertheless, consensus can be reached by cross-referencing studies employing different techniques and experimental protocols [43]. Through comparative analysis of cases and controls recurring patterns emerge, offering a comprehensive insight into gut microbiota dynamics [44].

### 3.1. Bacteria in IBD

*Escherichia coli (E. coli)* is a Gram-negative, facultative anaerobic bacterium commonly found in the lower intestine of warm-blooded organisms [45]. Elevated levels of *E. coli* have been observed in the IECs of patients with CD and UC [46]. Specifically, *Adherent-invasive E. coli (AIEC)* has been implicated in the early stages of IBD development [46]. *AIEC* can adhere to and traverse the intestinal mucosa and survive and multiply within macrophages, leading to the release of TNF, thereby increasing the permeability of the intestinal epithelium [46,47]. Additionally, it is associated with tissue damage resulting from the activation of the pro-inflammatory and tumorigenic transcription factor signal transducer and activator of transcription STAT3, along with the pro-inflammatory cytokine IL-17 [48]. Furthermore, *E. coli* has been shown to release colibactin, which damages DNA in IECs, potentially contributing to the increased susceptibility of IBD patients to CRC [49].

*Bacteroides fragilis (B. fragilis)* is a Gram-negative anaerobic bacterium that typically exists as a commensal in the gut microbiota [50]. Strains of *B. fragilis* expressing a zinc-dependent metalloprotease known as B. fragilis toxin (BFT or fragilysin) are referred to as *enterotoxigenic B. fragilis (ETBF)* [50]. *ETBF* acts as an opportunistic pathogen with pro-inflammatory properties that contribute to IBD [51]. BFT directly impacts signaling pathways such as Wnt, NF-κB, STAT3, and MAPK, resulting in elevated levels of Th17 cells, Tregs, and pro-inflammatory mediators, thereby increasing mucosal permeability [51]. Additionally, *ETBF* disrupts the colonic epithelial barrier by cleaving the zonula adherens protein E-cadherin [51]. Finally, *ETBF* induces the production of reactive oxygen species (ROS) and DNA damage by upregulating the expression of spermine oxidase in colonocytes [52].

Other bacteria, such as *Enterococcus faecalis* and *Streptococcus bovis*, have been implicated in promoting cytokine expression and inflammation in the colon, leading to the development of IBD [53,54]. *Enterococcus faecalis* is known for its production of damaging reactive oxygen species (ROS) [55]. Additionally, experimental models have shown that IL-10 knock-out mice are more susceptible to developing IBD when exposed to *Enterococcus faecalis* [56]. *Streptococcus bovis* is associated with the production of pro-inflammatory and pro-angiogenic cytokines, including IL-6, IL-8, and IL-17 [53].

*Fusobacterium nucleatum*, another pro-inflammatory bacterium, activates epithelial TLR4, which induces inflammation [57]. It has also been found to be abundant in the colonic mucosa of UC patients [58].

*Faecalibacterium prausnitzii (F. praunitzii)* and *Roseburia hominis* have garnered significant attention in recent years due to their status as butyrate-producing bacteria inhabiting the gastrointestinal tract [59]. A decline in their abundance has been noted in individuals with IBD [60]. In various in vivo models of chemically induced colitis, *F. prausnitzii* has been shown to mitigate the severity of intestinal inflammation by producing butyrate [61]. Butyrate helps to maintain a balance between Th17 and Tregs, promotes the production of anti-inflammatory cytokines such as interleukin IL-10, and inhibits NF-κB signaling as well as the production of IL-8, IL-12, and interferon-γ [62].

**Figure 3:**
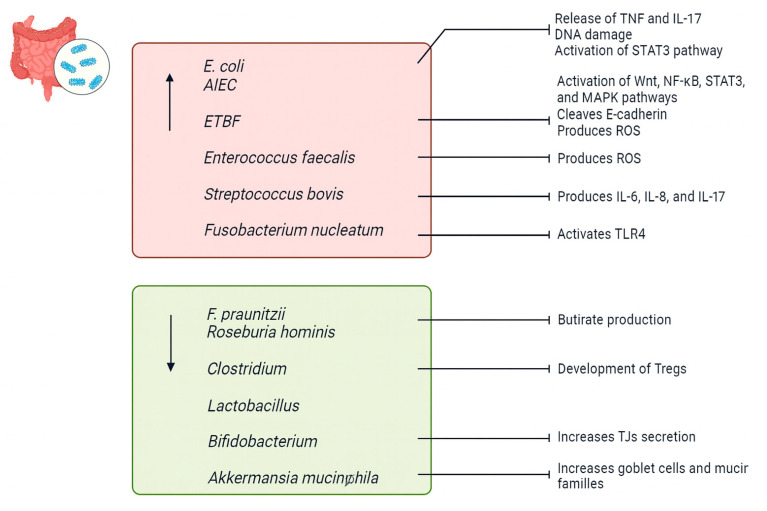
Diagram of bacteria increased and decreased in inflammatory bowel disease (IBD) along with their respective actions on the immune system.

Several other bacterial species within the *Clostridium*, *Lactobacillus*, and *Bifidobacterium* genera also appear to have beneficial effects against IBD [63,64]. For example, various strains of *Clostridia* can promote the development of Tregs in the colonic mucosa, potentially protecting against colitis [65]. *Bifidobacteria* have been shown to increase the secretion of TJs from intestinal cells, thereby improving symptoms in IBD mice with shortened intestines [66]. Last but not least, *Akkermansia muciniphila*, a mucophilic bacterium, can increase the numbers of goblet cells and mucin families in the intestinal epithelium, providing protection against IBD [67] (Figure 3).

### 3.2. Viruses in IBD

**Figure 4:**
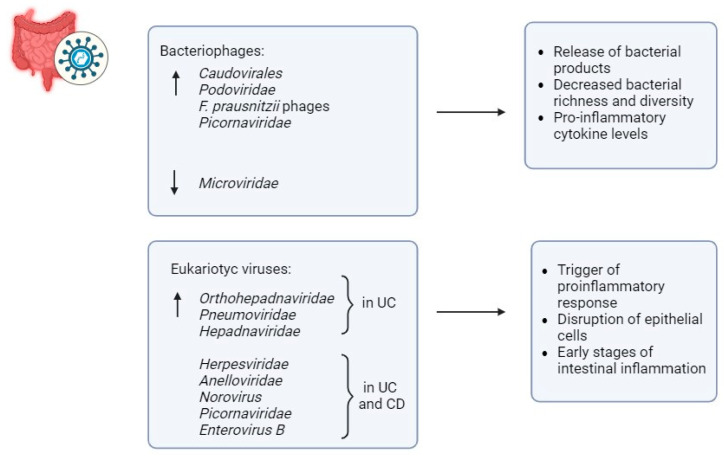
Diagram of viruses increased and decreased in inflammatory bowel disease (IBD) along with their respective actions on the intestinal environment and immune system.

Unlike bacterial diversity, increased virome diversity has been noted in IBD patients, suggesting the involvement of the gut virome in bacterial dysbiosis [68] (Figure 4). For their evolutionary characteristics, viruses can be classified into bacteriophages and eukaryotic-targeting viruses, both of which may possess single-stranded or double-stranded RNA or DNA genomes [69]. Bacteriophages typically have a direct influence on the makeup of bacteria, while eukaryotic viruses possess the ability to interact with not only human host cells but also other eukaryotic elements of the microbiota, like fungi [70]. Changes in the composition of the gut virome have been linked to the development and severity of IBD [69].

Specifically, metagenomics analysis has revealed an increase in bacteriophages of the family *Caudovirales* and a decrease in *Microviridaes* [69]. Elevated levels of *Caudovirales* have been observed in patients with UC and CD, with similar findings in gut biopsies of IBD patients compared to controls [71]. The virome has the potential to influence various elements within the microbiota, as demonstrated in Norman et al.’s research. This study highlighted the proliferation of *Caudovirales* bacteriophages in individuals with IBD compared to controls, alongside reduced bacterial richness and diversity, which are characteristic features of intestinal dysbiosis associated with IBD [71]. Increased viral diversity has also been correlated with gut dysbiosis and pro-inflammatory cytokine levels in mouse models [72]. Studies have reported the enrichment of bacteriophages Caudovirales and Podoviridae in mouse models of colitis [72]. Furthermore, elevated bacteriophage abundance has been linked to exacerbated colitis in germ-free mice [72]. Likewise, researchers have examined the interplay between bacteriophages and bacteria, focusing on *Faecalibacterium prausnitzii*, a bacterium typically diminished in individuals with IBD. The reduced presence of *F. prausnitzii* in IBD patients has been linked to a greater occurrence of *F. prausnitzii* phages compared to healthy controls, indicating increased phage-induced mortality of *F. prausnitzii* in IBD [73].

Eukaryotic viruses have also been linked to the initial phases of gut inflammation, hinting at a potential involvement in the development of IBD, supported by their capability to engage with host cells [69]. In this context, a greater presence of the eukaryotic *Orthohepadnaviridae* transcripts was observed in treatment-naive patients diagnosed early with UC compared to those with CD and healthy controls [74]. Furthermore, studies have observed an increased abundance of eukaryotic viruses such as *Pneumoviridae* and *Hepadnaviridae* in UC patients, and *Herpesviridae* in both CD and UC patients [74].

Likewise, research findings revealed a correlation between the prevalence of eukaryotic *Anelloviridae* in individuals diagnosed with IBD at a young age and those undergoing immunosuppressive therapy [75]. Moreover, research in mice with a CD risk gene, Atg16L1HM, has shown that *Norovirus* can induce intestinal pathologies [76]. Similarly, murine *Norovirus* has been found to induce colitis in an IL10-deficient mouse model of IBD in a microbiota-dependent manner [76].

Viruses play a role in damaging the gut barrier through various mechanisms [77]. Phages indirectly trigger the immune response by releasing bacterial products when bacteria break down or move across the epithelium (transcytosis), which activates pattern recognition receptors on intestinal epithelial cells or resident immune cells [77]. Furthermore, specific elements from the virome can disrupt barrier function and influence gut health, often by interacting with other microorganisms that inhabit the gut [78]. For example, Sinha et al. gathered viral-like particles (VLPs) from three UC patients, primarily containing *Microviridae* phages, along with crAss-like, *Siphoviridae*, and *Podoviridae* phages to a lesser extent, and introduced them into mice with human-like gut microbiota [79].

UC VLP transplantation exacerbated colitis severity [79]. Similarly, Adiliaghdam et al. discovered that the virome of healthy individuals directly triggered unusual anti-inflammatory innate immune responses, whereas viromes obtained from UC and CD patients, primarily containing *Picornaviridae* and *Enterovirus B*, caused inflammation. This inflammatory response was effectively reversed by viromes from non-IBD individuals [80].

Additional research has highlighted the influence of viruses on innate immune responses. For example, filamentous Pf bacteriophages derived from *Pseudomonas aeruginosa* are taken up by DCs, macrophages, and B-cells, triggering type-I interferon responses that facilitate infection by related bacteria [81].

Interestingly, a recent investigation revealed that enteric viral infections promote the proliferation of distinct immune cell subsets within the intestinal tract, including colonic and small intestinal lamina propria leukocytes such as effector memory T-cells, macrophages, and plasmacytoid Dcs [82].

Notwithstanding these findings, exploration of the virome’s influence on intestinal immunity and barrier functions is still in its nascent stages. Besides identifying virome dysbiosis in inflammatory bowel disease (IBD), there remains a scarcity of studies detailing virome-induced pathological occurrences in the intestinal mucosa. Despite their persuasiveness, these studies require additional verification, primarily due to the constraints posed by interindividual and intercohort variabilities, which limit the applicability of their findings.

### 3.3. Archaea in IBD

**Figure 5:**
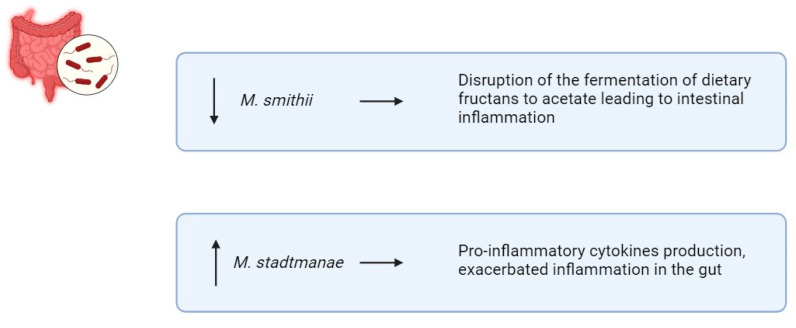
Diagram of archaea increased and decreased in inflammatory bowel disease (IBD) along with their respective actions.

Archaea are single-celled prokaryotes which are like bacteria but genetically closer to eukaryotes [68]. The predominant archaea in the human gut are methanogens, notably *Methanobrevibacter* and *Methanosphaera* [68]. Particularly, methanogens such as *Methanobrevibacter smithii (M. smithii)* and *Methanosphaera stadtmanae (M. stadtmanae)* play a role in IBD pathogenesis by influencing immune system dysregulation [69] (Figure 5). Decreased levels of *M. smithii* have been observed in individuals with IBD [70]. *M. smithii* contributes to digestive health by facilitating the fermentation of dietary fructans to acetate, which helps maintain gut homeostasis [70]. Reductions in *M. smithii* abundance may disrupt this process, leading to intestinal inflammation and contributing to IBD progression [70]. Conversely, studies have shown that the abundance of *M. stadtmanae* increases up to threefold in IBD patients [71]. This archaeon stimulates DCs to produce pro-inflammatory cytokines, exacerbating inflammation in the gut [71]. The elevated presence of *M. stadtmanae* may contribute to the dysregulation of the immune system observed in IBD [71]. Additionally, halophilic archaea may also play a role in IBD etiology [72]. However, Chehoud et al. reported no significant alterations in the archaeome associated with IBD [73]. Nevertheless, their contribution to IBD onset and progression remains contentious, likely due to insufficient research.

### 3.4. Fungi in IBD

**Figure 6:**
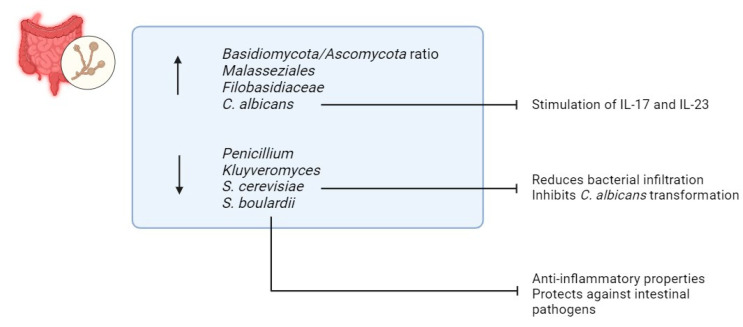
Diagram of fungi increased and decreased in inflammatory bowel disease (IBD) along with their respective actions.

Fungi constitute a small fraction, only 0.1%, of the overall microbial community in the gastrointestinal tract [74]. However, despite their low abundance, alterations in the mycobiome have been found to be closely associated with IBD [75] (Figure 6). Studies consistently report reduced fungal diversity in individuals with IBD, indicating a potential role of fungi in the pathogenesis of the disease [75,76,77]. Recent research conducted by Sokol et al. has shed light on specific changes in the mycobiome of IBD patients, revealing an elevated ratio of *Basidiomycota/Ascomycota* [75]. Additionally, heightened levels of *Malasseziales* and *Filobasidiaceae*, along with diminished levels of *Penicillium* and *Kluyveromyces*, have been documented in IBD patients, further highlighting the intricate relationship between fungal dysbiosis and IBD pathophysiology [75].

Among the various fungal species, *Candida* has garnered particular attention due to its increased prevalence in individuals with IBD [73,75]. Colonization by *Candida albicans* has been implicated in exacerbating inflammation through the stimulation of pro-inflammatory cytokines such as IL-17 and IL-23 [78]. This dysregulated immune response contributes to the chronic inflammation characteristic of IBD [78].

Conversely, reduced levels of *Saccharomyces cerevisiae (S. cerevisiae)* have been observed in IBD patients [75]. *S. cerevisiae* appears to play a protective role against IBD by inhibiting the binding of *AIEC* to intestinal mucosa, thereby reducing bacterial infiltration and subsequent inflammation [79]. Moreover, *S. cerevisiae* inhibits the transformation of *C. albicans* into its invasive hyphal form, a process mediated by the blockade of aspartyl proteases 2 and 6 [79,80]. By preventing fungal invasion and dysbiosis, *S. cerevisiae* contributes to maintaining intestinal homeostasis and reducing the risk of IBD development or exacerbation [79,80].

Furthermore, *Saccharomyces boulardii*, a probiotic yeast, has emerged as a potential preventive agent against IBD [81,82]. Its anti-inflammatory properties and ability to protect against intestinal pathogens make it a promising candidate for adjunctive therapy in IBD management [81,82].

### 3.5. Microbiota-Derived Metabolites Involved in IBD

#### 3.5.1. Short-Chain Fatty Acids (SCFAs)

SCFAs, produced by intestinal commensal bacteria through the fermentation of dietary fiber or other indigestible carbohydrates, play a pivotal role in maintaining intestinal health [83]. They accomplish this by modulating luminal pH, enhancing mucus production, serving as an energy source for IECs, and fortifying mucosal immune function, resulting in anti-inflammatory effects through the regulation of colonic Tregs [83]. The three primary SCFAs derived from the microbiota are acetate, propionate, and butyrate, with a ratio of approximately 3:1:1 [83]. Acetate is generated by various gut microbes, propionate is primarily produced by *Bacteroidetes*, *Negativicutes*, and *Lachnospiraceae*, while butyrate is predominantly synthesized by *Eubacterium*, *Clostridium*, and *Fusobacterium* [83].

SCFAs modulate cellular functions by interacting with G protein-coupled receptors expressed in IECs, including the orphan G protein-coupled receptor (GPR43) [84,85]. Binding of SCFAs to GPR43 mitigates inflammation, as evidenced by the heightened susceptibility to colitis observed in GPR43-deficient mice models [83,84].

**Figure 7:**
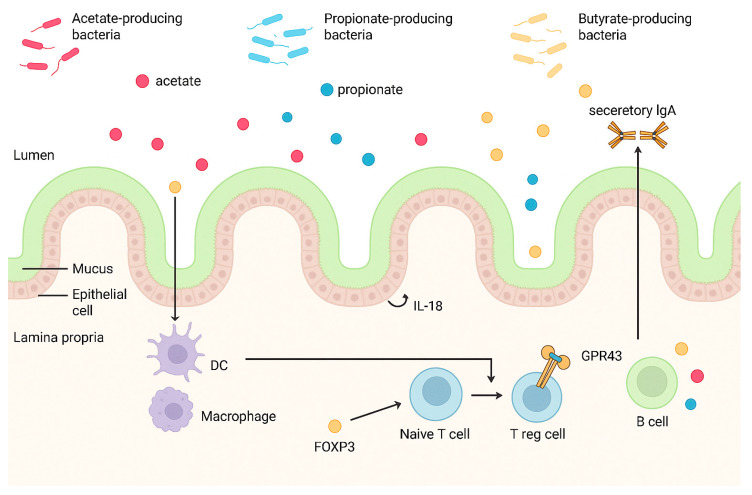
Short-chain fatty acids (SCFAs) and their effects on the host.

Furthermore, SCFAs regulate intestinal homeostasis by stimulating the production of antimicrobial peptides and intestinal IgA, as well as promoting epithelial homeostasis through IL-18 production [86,87]. They also inhibit the expression of NF-κB and the secretion of TNF-α, while exerting anti-proliferative effects and exhibiting [85,86,87] (Figure 7).

Numerous studies have consistently reported lower levels of SCFAs in the feces of IBD patients compared to healthy individuals, indicating a potential involvement of decreased concentrations of SCFA-producing bacteria and SCFAs in the chronic intestinal inflammation and pathophysiology of IBD [84]. Additionally, research by Hu et al. has demonstrated the loss of butyrate-producing bacterial species, such as *F. prausnitzii*, *Roseburia hominis*, and *Clostridium clusters IV* and *XIVa*, as evidenced by reduced fecal butyrate levels in IBD patients [88]. This decrease in SCFA-producing bacteria is often accompanied by an increase in pathogenic bacteria like *Escherichia-Shigella*, which degrade SCFAs to counteract their anti-inflammatory effects, ultimately contributing to immune dysregulation in the intestinal tract of individuals with IBD [88].

#### 3.5.2. Bile Acids (BAs)

BAs, a type of steroid acid present in bile and synthesized from cholesterol by the liver, play a crucial role in the emulsification and absorption of fats, as well as in the elimination of cholesterol [89]. They exert metabolic effects by binding to various receptors, including the farnesoid X receptor (FXR), pregnane X receptor (PXR), transmembrane G protein-coupled receptor 5 (TGR5), vitamin D receptor, and androstane [89].

There exists a bi-directional relationship between BAs and the microbiota [90]. In the intestine, the microbiota converts primary BAs, originating from the liver, into secondary BAs [90]. Conjugated BAs excreted in bile are partly deconjugated, dehydroxylated, and reduced [91,92]. Deconjugation of BAs, achieved by removing glycine and taurine, prevents their reuptake in the small intestine by the ASBT transporter [91,92]. This process relies on the activity of intestinal bacteria possessing the Bile Salt Hydrolase (BSH) enzyme, such as *Lactobacilli* and *Bifidobacteria* [91,92]. Deconjugated BAs not absorbed by the ileum via ASBT reach the colon, where they undergo dehydroxylation, generating the secondary BAs lithocholic acid and deoxycholic acid [91,92]. This dehydroxylation process involves several reactions conducted by bacteria belonging to the *Clostridium (clusters XIVa* and *XI)* and *Eubacterium* genera, which are part of the *Firmicutes* phylum [91,92]. Additionally, in humans the microbiota can generate secondary BAs through isomerization reactions, with ursodeoxycholic acid formed by the isomerization of chenodeoxycholic acid by *Clostridium absonum* [91,92].

BAs possess potent antimicrobial properties that may alter gut microbiota composition and density [93]. They exert direct antimicrobial effects on bacteria like *Bifidobacterium breve* and *Lactobacillus salivarius*, while their indirect effects involve stimulating the production of antimicrobial peptides from the host and activating FXR [93]. Activation of the nuclear receptor FXR has been shown to influence the composition of the microbiota [94]. Mice lacking FXR expression (Fxr-/-) and fed a high-fat diet exhibit an increase in the percentage of *Firmicutes* and a corresponding decrease in the percentage of *Bacteroidetes* compared to wild-type mice fed the same diet [95]. The accumulation of primary BAs in FXR-deficient mice suggests that the microbiota may have limited ability to metabolize BAs in the absence of this receptor [95].

Reduced levels of BAs in the intestinal lumen promote the proliferation of Gram-negative bacteria, including several pathogens [96]. Additionally, decreased levels of fecal BAs are associated with an increased presence of potentially pathogenic bacteria capable of inducing inflammation, such as *Enterobacteriaceae* [96]. Conversely, elevated levels of BAs in the intestine promote Gram-positive bacteria belonging to the *Firmicutes* phylum [96].

Numerous studies have reported altered bile acid profiles in fecal samples of IBD patients [97]. Concomitantly, studies have shown a significant reduction in FXR expression in CD patients [98]. Moreover, Labbé et al., analyzing metagenomics samples from the Human Microbiome Project and MetaHit, found a reduction in clusters of BSH genes associated with *Firmicutes* in IBD [99]. Among IBD patients, dysbiosis seems to lead to a lack of secondary BAs in the gut, and the beneficial effects of SBA supplementation on intestinal inflammation have been validated in animal models, possibly due to SBAs inhibiting Th17 cell function [97].

#### 3.5.3. Bacterial Self-Metabolites

Tryptophan, an essential aromatic amino acid, undergoes significant alterations in concentration and metabolism in individuals with IBD, including changes in the activity of associated enzymes [100]. Dietary tryptophan follows the following three metabolic pathways: the kynurenine pathway, serotonin pathway, and indole pathway [100]. Microbial metabolism of tryptophan within the indole pathway yields various bioactive indole derivatives serving as agonists for the Aryl hydrocarbon receptor (AhR), a critical transcription factor regulating T-cell immunity, cytokine expression, and anti-inflammatory effects via IL-22 [100]. Consistent with these findings, reduced AhR expression was noted in the inflamed mucosa of CD patients [100]. Certain *Lactobacillus* strains capable of AhR activation have demonstrated a reduction in the severity of DSS-induced colitis [101]. Critical research has identified a significant association between tryptophan metabolism and IBD in clinical cohorts, indicating an inverse relationship between tryptophan levels and IBD severity. Similar findings in mouse models suggest that tryptophan deficiency may exacerbate colitis.

Succinate, a tricarboxylic acid (TCA) intermediate produced by both the host and the microbiota, has garnered attention due to its potential link with IBD [102]. Elevated succinate levels act as a pro-inflammatory signal, particularly increased in CD patients [102]. Fecal succinate levels are also heightened in both UC and CD patients [102]. Furthermore, reduced abundances of succinate-utilizing *Phascolarctobacterium* are observed in UC and CD patients compared to healthy individuals [102].

Histamine, which contributes to abdominal pain in patients with IBD, is predominantly produced by *Klebsiella aerogenes*, which is highly abundant in the fecal microbiota of IBD patients [103]. Elevated levels of histamine suppress the expression of tight junction and MUC2 proteins, diminish intestinal autophagy, and impair the function of colonic goblet cells in mucus secretion, ultimately resulting in the compromised integrity of the intestinal mucosal barrier [103].

Desulfovibrio, a prominent genus of sulfate-reducing bacteria (SRB), can induce sulfide production, leading to symptoms such as frequent defecation, weight loss, and heightened intestinal permeability [104]. Consequently, individuals with UC typically exhibit elevated hydrogen sulfide levels in the intestines [104].

Finally, self-metabolites of bacteria, such as colibactin and indoleamine, have DNA damaging effects on epithelial cells and confer an increased risk of CRC [105].

#### 3.5.4. Vitamins

Vitamin synthesis represents a critical metabolic function facilitated by the gut microbiota [106]. *Clostridium* is involved in synthesizing folate, cobalamin, niacin, and thiamine [106]. *Bifidobacteria* contributes to folate synthesis, while *Bacteroides* is implicated in producing riboflavin, niacin, pantothenate, and pyridoxine [106]. Certain intestinal bacteria rely heavily on host-supplied vitamins, suggesting that vitamin deficiency could impact bacterial growth or microbial utilization of host vitamins [106]. Inadequate dietary vitamin K disrupts the microbial community, leading to impaired blood clotting. This suggests a potential association between vitamin K deficiency and intestinal bleeding symptoms in IBD. While vitamin K1 is primarily obtained from food, vitamin K2 is synthesized by gut bacteria [107]. In neonates or healthy individuals, *E. coli* helps create an anaerobic environment in the intestine, facilitating the colonization of other anaerobes and vitamin K production to resist pathogenic bacteria invasion [107]. Additionally, vitamin K2 promotes the abundance of SCFAs and SCFA-producing genera in the colon [107].

Finally, while gut microbiota do not directly synthesize vitamin D, it significantly influences its metabolism and absorption [108]. Specific gut bacteria regulate the expression and activity of enzymes responsible for converting vitamin D to its active form, calcitriol, which plays vital roles in calcium and phosphorus metabolism, immune modulation, and anti-inflammatory actions [108]. Moreover, certain gut bacteria enhance the expression of genes involved in vitamin D metabolism, thereby promoting its bioavailability and activity [108]. This intricate interplay underscores the crucial role of gut microbiota in maintaining vitamin D homeostasis and its potential impact on overall health [108]. Dysbiosis, or the disruption of gut microbiota composition, may compromise vitamin D absorption and metabolism, potentially leading to vitamin D deficiency [108].

## 4. Manipulation of Microbiota as a Treatment Strategy in IBD

A growing body of evidence supports the therapeutic potential of microbiota-directed interventions in IBD. These approaches aim to restore microbial equilibrium, reinforce epithelial barrier function, and modulate host immune responses (Table 1).

### 4.1. Diet

A Western-style diet, characterized by elevated levels of protein, fat, and sugar coupled with low fiber intake, has been associated with an augmented risk of IBD [122]. This dietary pattern has been demonstrated to diminish microbial diversity and compromise the integrity of the colonic mucus layer, facilitating the proliferation and heightened activity of pathogenic bacteria [122]. Consequently, this dysbiosis contributes to the accumulation of specific immune cell populations and disrupts the normal absorptive function of enterocytes [122]. Clinical investigations involving patients with UC who transitioned to a low-fat, high-fiber diet revealed a reduction in the relative abundance of *Actinobacteria* and an augmentation in *F. prausnitzii* levels [122]. Additionally, there was an increase in the concentration of anti-inflammatory metabolites such as acetate in their fecal matter [122].

Moreover, studies have highlighted a prevalent deficiency in vitamin D among individuals with IBD, with lower expression levels of the vitamin D receptor (VDR) in the intestines correlating with heightened inflammation severity [123]. The VDR pathway emerges as a promising therapeutic target for mitigating diet-induced inflammatory bowel disease [123]. Vitamin D has been shown to exert a beneficial impact on the gut microbiota composition in IBD patients, fostering the proliferation of beneficial bacterial species including *Roseburia, Alistipes, Parabacteroides*, and *Faecalibacterium*, while suppressing the abundance of pathogenic bacteria like *Ruminococcus gnavus* [123]. However, these effects appear to be transient, suggesting that sustained maintenance of this favorable microbial balance may necessitate additional interventions beyond long-term vitamin D supplementation alone [123].

Diet has garnered growing interest as a readily modifiable environmental factor, presenting potential as both a preventive measure and treatment option for IBD.

#### 4.1.1. The Crohn’s Disease Exclusion Diet (CDED)

The CDED is a structured, whole-food-based dietary therapy designed to reduce exposure to specific dietary components that are thought to contribute to intestinal inflammation through detrimental effects on the gut microbiota, mucosal immunity, and barrier function [118]. It is implemented in the following three progressive phases: an initial strict phase (weeks 0–6) that eliminates key pro-inflammatory food groups and additives (such as emulsifiers and maltodextrins), followed by gradual reintroduction of selected foods (weeks 6–12), and finally a maintenance phase tailored to long-term adherence. Unlike exclusive enteral nutrition (EEN), CDED allows for the consumption of solid foods and has been shown to improve tolerance, compliance, and patient quality of life [118].

Mechanistically, CDED promotes favorable alterations in gut microbial composition and function [118]. Clinical and microbiome studies have demonstrated that remission with CDED is consistently associated with a reduction in Proteobacteria—particularly pathogenic *Escherichia coli*—and a concomitant expansion of beneficial Firmicutes, including short-chain fatty acid (SCFA) producers [118]. Although overall microbial diversity does not fully normalize, the functional profile of the microbiota improves, characterized by an increased capacity for SCFA production and a decreased expression of inflammatory metabolites such as kynurenine. These microbial and metabolic shifts are thought to contribute to restoring epithelial barrier integrity and reducing mucosal inflammation. Notably, sustained remission with CDED has been linked to persistent changes in tryptophan metabolism, favoring serotonin and indole pathways over pro-inflammatory kynurenine production, underscoring the diet’s capacity to modulate host–microbiome interactions at multiple levels.

Growing evidence from randomized trials and real-world data supports the efficacy of CDED, particularly in pediatric patients with mild-to-moderate luminal CD [119]. When combined with partial enteral nutrition (PEN), CDED has shown superior long-term remission rates compared to EEN, followed by free diet [120]. While evidence in adult and severe CD phenotypes is still limited, CDED represents a promising, mechanistically grounded intervention that aligns dietary modulation with disease control, especially when used early in the disease course or in well-selected patient subgroups [121].

#### 4.1.2. The Specific Carbohydrate Diet (SCD)

The SCD, initially developed in the 1920s for celiac disease, has been explored as a potential treatment for IBD due to its success in managing UC [124]. The diet involves eliminating complex carbohydrates, which can ferment in the colon and promote inflammation in IBD, while allowing simple sugars [125]. Allowed foods include unprocessed meats, certain vegetables and fruits, fats, aged cheeses, and lactose-free yogurt, while prohibited foods include milk, grains, soft cheeses, and non-honey sweeteners. Reintroduction of prohibited foods occurs gradually. Studies have shown promising results, with a significant portion of patients experiencing clinical remission and even discontinuation of corticosteroid therapy [126]. A case study involving a young woman diagnosed with UC who followed the SCD demonstrated a significant improvement in UC symptoms and notable changes in her microbiome [127]. Before starting the SCD, the predominant bacterial species in her microbiota were *Fusobacterium ulcerans* and *Viellonella dispar*. In contrast, healthy individuals without dietary restrictions showed a different bacterial composition, with the dominant species being *Bacteriodeaceae*, *Ruminococcaceae*, and *Lachnospiraceae* and the absence of *Fusobacterium ulcerans* and *Viellonella dispar*. Following two weeks on the SCD, the patient’s microbiome exhibited a decrease in *Fusobacterium ulcerans* and a substantial increase in various *Enterobacteriaceae* species [127]. In another study, six individuals with CD were compared to two healthy controls following either the SCD or a low-residue diet for thirty days [128]. Analysis of fecal samples collected at baseline and day 30 revealed a reduced microbial diversity in CD patients initially. Following the SCD, there was an increase in microbial diversity characterized by a higher prevalence of nonpathogenic species within the clostridia family. However, despite these changes, there was no clinically significant improvement observed in the patients’ conditions. Data on SCD’s efficacy, especially in adults, are limited, and further research, possibly through comparative case–control studies, is needed to better understand its impact on the microbiota and overall efficacy in managing IBD.

#### 4.1.3. The Low-FODMAP Diet

The low-FODMAP diet, like the SCD, involves reducing poorly absorbed and highly fermentable carbohydrates [129]. Whilst SCD favors monosaccharides, low FODMAP discourages their intake. Both diets aim to alleviate dysbiosis, inflammation, and other symptoms by limiting certain carbohydrates. High-FODMAP foods like high-lactose dairy, excess fructose vegetables/fruits, and food rich in fructans/galactans and polyols are excluded. Low-FODMAPs foods such as dairy free from lactose, low fructans and galactans from vegetables, and low fructose are allowed. Initially strict for 4-6 weeks, the diet gradually reintroduces FODMAPs while monitoring symptoms [130]. Supervision by a dietitian is recommended to prevent nutritional deficiencies. Studies show the low-FODMAP diet improves IBD patients’ quality of life and reduces IBS-like symptoms, which is significant as over 30% of IBD patients also have concurrent IBS [131,132]. In a small randomized, controlled, crossover trial involving quiescent CD patients, the low-FODMAP diet led to improvement in overall gastrointestinal symptoms [133]. Nine patients underwent randomization to either low- or high-FODMAP diets for 21 days with a ≥21-day washout period in between. Fecal samples collected at the end of each diet period showed no significant changes in SCFAs, pH, or total bacterial abundance. However, the relative abundance of butyrate-producing Clostridium cluster XIVa and mucus-associated Akkermansia muciniphila increased, while Ruminococcus torques decreased during the high- compared to the low-FODMAP diet. No effects were observed in calprotectin levels, but symptom severity worsened with the high-FODMAP diet. Despite the acceptance of treating IBD patients’ IBS-like symptoms with a low-FODMAP approach, its impact on underlying inflammation remains poorly understood and needs further research studies.

#### 4.1.4. The Gluten-Free Diet

The gluten-free diet, primarily used to manage celiac disease by eliminating gliadin-containing foods, has also been adopted by individuals with non-celiac gluten sensitivity who experience relief from IBS-like symptoms upon gluten elimination despite lacking celiac disease markers [134]. The potential benefits of this diet for IBD patients remain unclear, although it may involve immune system inactivation by wheat proteins and reduced intestinal permeability. Additionally, the gluten-free diet typically involves low-FODMAP consumption, potentially offering similar benefits to the low-FODMAP approach. However, its implementation requires supervision due to potential micronutrient and dietary fiber deficiencies. Studies have shown varying results regarding the efficacy of the gluten-free diet in improving IBD symptoms [135,136,137]. While some report symptom improvement, others find no significant differences in disease activity, hospitalization, or surgery rates between those adhering to the diet and those who do not. Furthermore, differences in microbiota composition have been observed between CD patients on a gluten-free diet and those on a regular diet, suggesting potential dietary influences on gut microbiota diversity, especially regarding phyla *Bacteroidetes* and *Firmicutes*. However, such differences were not consistently observed in UC patients.

#### 4.1.5. The Anti-Inflammatory Diet (AID)

The AID aims to reduce inflammation by incorporating foods rich in anti-inflammatory phytonutrients, spices, and omega-3 polyunsaturated fatty acids from fish. Daily consumption of fruits and vegetables is encouraged to provide essential anti-inflammatory compounds such as vitamins B3, B6, E, C, beta-carotene, zinc, and magnesium. While animal proteins are permitted, plant proteins from legumes are preferred. A practical application of this diet for IBD patients known as the Nutritional Regimen for IBD (IBD-AID) was introduced by Olendzki et al. [138]. Unlike the SCD, the IBD-AID allows for the inclusion of some grains, gluten, and probiotic foods to address deficiencies observed in the SCD. Additionally, it emphasizes the consumption of omega-3 fatty acids while reducing total and saturated fats. The IBD-AID consists of four phases with varying food categories and textures. A study conducted by Olendzki et al. involving 11 IBD patients refractory to pharmacological treatment or experiencing inadequately controlled symptoms demonstrated symptom improvement and medication reduction in all participants [138]. Keshteli AH et al. designed a 6 month, open-label, randomized, placebo-controlled trial in adult UC patients [139]. Patients in the AID group showed a higher subclinical response and increased levels of fecal *Bifidobacteriaceae*, *Lachnospiraceae*, and *Ruminococcaceae*. However, there is currently limited data available regarding inflammatory markers or microbiome modifications associated with this diet.

#### 4.1.6. The Mediterranean Diet

The Mediterranean diet shares similarities with the anti-inflammatory diet, emphasizing the consumption of phytonutrients, unsaturated fats like olive oil, omega-3 polyunsaturated fats, vegetables, whole grains, nuts, and limited red meat intake. Adherence to this diet has been linked to reduced inflammatory markers [140]. Evidence suggests its potential effectiveness in managing IBD, with pre-illness dietary habits indicating that high fruit and fiber intake may protect against CD, while a high vegetable intake may prevent UC. Conversely, high meat, omega-6 fatty acids, polyunsaturated fatty acids, and total fat intake are associated with increased CD and UC incidence. Unlike other dietary approaches, the Mediterranean diet is less likely to lead to nutritional deficiencies [141]. Clinical and translational research on the Mediterranean diet suggests its promising use in managing IBD, with potential for additional insights through further studies. One study involving 153 healthy Italian subjects found that high adherence to the Mediterranean diet positively influenced gut microbiota and associated metabolome [142]. Another study with eight CD patients following the Mediterranean diet for six weeks showed significant changes in gene expression and a trend toward normalization of the intestinal microbiota with an increase in the expression of *Bacteroidetes*, *Clostridium cluster IV*, and *Clostridium cluster XIVa* and a decrease in the abundance of *Proteobacteria* and *Bacillaceae* [143]. While the Mediterranean diet’s high fiber content may be unsuitable during disease flares, it is recommended post-remission with appropriate adjustments. Foods like pulses promote prebiotic effects, while vegetables, especially broccoli, may prevent CD relapse. Fruits, when treated with a juice extractor, retain immunomodulatory minerals and vitamins, along with olive oil and bluefish, which have anti-inflammatory effects.

### 4.2. Prebiotics

Prebiotics are non-digestible dietary fibers that are selectively fermented by the gut microflora and serve as fuel for beneficial bacteria in the gut [144]. Prebiotics mostly include fructooligosaccharides (FOSs), galactooligosaccharides (GOSs), and other oligosaccharides, such as pectin [144]. They can help promote the growth and activity of these beneficial microbes, which in turn can have positive effects on gut health [144]. In patients with IBD, the manipulation of the gut microbiota using prebiotics has shown promise as a therapeutic approach [144].

Many studies have shown that prebiotic supplementation may improve symptoms and quality of life in patients with IBD [145]. This may be due to the positive effects of prebiotics on gut microbial composition and function [146]. Prebiotics such as inulin have been shown to induce the growth of SCFA-producing bacteria, including *Lactobacillus*, *F. prausnitzii*, and *Bifidobacterium* [145]. Inulin has also been shown to improve histological lesions in patients with pouchitis [147]. FOSs are known to increase the population of endogenous microflora, particularly *Lactobacillus* and *Bifidobacterium* [148]. FOSs and GOSs can improve the levels of *F. prausnitzii* [148]. By promoting the growth of these microbes, prebiotics can help restore microbial balance in the gut, which is often disrupted in patients with IBD [148]. When beneficial bacteria ferment prebiotics they produce SCFAs such as acetate, propionate, and butyrate which have anti-inflammatory properties and help maintain gut barrier function [149,150].

While prebiotics show promise as a complementary therapy for IBD, they are typically used in conjunction with other treatments such as medication and dietary modifications [151]. The effectiveness of prebiotics may vary depending on factors such as the type of prebiotic used, the dosage, and individual differences in gut microbiota composition.

Overall, the manipulation of the gut microbiota by prebiotics represents a promising approach for managing IBD. However, more research is needed to fully understand the mechanisms of action and the optimal use of prebiotics in this context. Specifically, given that prebiotics are fibers, albeit soluble, it is crucial to acknowledge the potential for poor tolerance among individuals with stricturing CD.

### 4.3. Probiotics

Probiotics, defined as live microorganisms offering various health benefits when consumed in adequate amounts, have emerged as a potential therapeutic option for IBD [109], (Table 2). The rationale behind probiotic therapy for IBD lies in its ability to modulate gut microbiota composition, enhance intestinal barrier function, inhibit the colonization of pathogenic microbes, and regulate local and systemic immune responses [110]. Their actions, dependent on type, dose, and host interactions, range from direct antibacterial effects through substance production to non-immunological actions like nutrient competition, increased mucus production, pH alteration, tight junction formation, and tissue repair [110]. Additionally, probiotics modulate the immunological response by influencing immunoglobulin and cytokine production, regulating the NF-κB pathway, and balancing pro-inflammatory and anti-inflammatory cytokines like IL-8, TNF-α, IFN-γ, IL-10, and TGF-β [152].

Modulating the gut microbiota through probiotics remains a promising approach; however, recent evidence suggests that their clinical efficacy in IBD is limited and highly strain-dependent [153]. Numerous studies report inconsistent outcomes, particularly in Crohn’s disease, with more encouraging results observed in ulcerative colitis. The therapeutic effect of probiotics appears most effective when specific strains are targeted to well-defined patient phenotypes or used as adjunctive therapy. Recent meta-analyses underscore the need for improved strain selection, optimized dosing, and a deeper understanding of underlying mechanisms [154].

Although no standardized guidelines currently exist for probiotic dosing, most commercially available formulations provide between one and ten billion colony-forming units (CFUs) per dose [155]. Certain probiotics, such as *Saccharomyces boulardii, Escherichia coli Nissle 1917,* and *Bifidobacterium breve* strain Yakult, have demonstrated efficacy and safety comparable to mesalamine in maintaining clinical remission in UC patients as assessed through quality of life measures, endoscopy, and histology [82,156,157]. Notably, European Crohn’s and Colitis Organization (ECCO) guidelines endorse *E. coli Nissle 1917* as a viable alternative to mesalamine for UC remission maintenance [158]. Moreover, when combined with conventional medications like mesalamine, *Lactobacillus reuteri ATCC 55730* has shown improved clinical response and remission rates in children with UC [159]. *Clostridium butyricum (C. butyricum)* has also shown effectiveness in suppressing inflammation in experimental colitis and preventing pouchitis in UC patients [160]. Additionally, *Saccharomyces boulardii* has been shown to reduce recurrence rates in CD patients when combined with mesalazine [161]. This yeast strain can enhance intestinal barrier function by reducing intestinal permeability, increasing plasma levels of the anti-inflammatory cytokine IL-10 and intestinal IgA secretion, and preventing relapses in CD patients [161]. Finally, *Lactobacillus plantarum* has been found by Jin et al. to restore gut barrier function and reduce intestinal inflammation in a mouse model of DSS-induced colitis [162].

Various probiotic cocktails have been proposed for IBD treatment [162]. De Simone formulation (DSF) is a mixture of eight bacterial strains, including *Lactobacillus acidophilus, L. plantarum, L. casei, L. delbrueckii subspecies bulgaricus, Bifidobacterium breve, B. longum, B. infantis,* and *Streptococcus salivarius* subspecies thermophiles [163]. Studies have shown its effectiveness in inducing remission in patients with mild-to-moderately active UC, preventing or maintaining remission in chronic pouchitis following ileal pouch-anal anastomosis for UC with lower incidence rates of acute pouchitis and higher maintenance of antibiotic-induced pouchitis remission compared to the placebo group, and preventing endoscopic recurrence after surgery for CD [164,165]. Moreover, there appears to be a synergistic effect between DSF and conventional drugs, with potential mechanisms including the enhancement of the anti-inflammatory effects of mesalazine, inhibition of free radical production, and suppression of leukotriene and IL-1 production [166]. Combining DSF with standard therapy has been proven to improve histological scores in children with UC [167]. Another study by Miele et al. showed that combining DSF with mesalamine and steroids could significantly improve the remission rate in children with UC [168].

The use of a combination of *L. acidophilus*, *L. plantarum*, *B. lactis*, and *B. breve* has been proven to boost the production of intestinal mucus and goblet cells in mice [158]. Similarly, another mixture containing *L. plantarum*, *L. acidophilus*, *L. rhamnosus*, and *E. faecium* has been shown to promote wound healing and strengthen the integrity of tight junctions between epithelial cells [159]. For patients with UC, combining mesalazine with a probiotic mixture of *L. salivarius*, *L. acidophilus*, and *B. bifidum* strain demonstrated beneficial effects, resulting in a shorter recovery time, lower disease activity, and improved endoscopic images [160]. Finally, Chen et al. reported that the probiotic mixture of *B. infantis*, *L. acidophilus,* and *E. faecalis* with or without *Bacillus cereus* could restore the relative abundance of *Lactobacillus*, *Bifidobacterium*, *Bacteroides,* and *Akkermansia* in a mouse model of DSS-induced chronic colitis [161].

Probiotic supplementation can not only restore intestinal microbiota depletion but also potentiate medication effectiveness. For example, the co-administration of *Bifidobacterium* with mesalazine not only ameliorates IBD symptoms but also reduces adverse effects [169]. Thus, prudent exploitation of the benefits conferred by gut flora may yield a synergistic effect greater than the mere sum of individual components.

The above-mentioned probiotic strains, among others, offer promising avenues for managing IBD by modulating gut microbiota, reducing inflammation, and maintaining intestinal barrier function. However, while some studies have reported positive outcomes, further research is needed to elucidate the optimal dosing, duration, and efficacy of probiotics in the context of IBD treatment. Additionally, individual responses to probiotic therapy may vary, highlighting the importance of personalized approaches and continued investigation in this field.

**Table 2:** Summary of probiotic interventions in inflammatory bowel disease.

**Probiotic or Mixture**	**Effect**	**Source**	**Reference**
*Saccharomyces boulardii*	Reduces recurrence in CD, improves barrier function, ↑ IL-10, and ↑ IgA	Human studies (CD patients) and experimental data	[153]
*Escherichia coli Nissle 1917*	Alternative to mesalamine in UC remission (ECCO recommendation)	Endorsed by ECCO guidelines	[109]
*Bifidobacterium breve (Yakult)*	Comparable efficacy to mesalamine in UC remission maintenance	UC patients—quality of life, endoscopy, and histology	[151]
*Lactobacillus reuteri ATCC 55730*	↑ remission in children with UC when combined with mesalamine	Pediatric UC study with mesalamine	[110]
*Clostridium butyricum*	Suppresses colitis inflammation and prevents pouchitis in UC	Experimental colitis and clinical pouchitis studies	[152]
*Lactobacillus plantarum*	Restores barrier function and ↓ inflammation (DSS-induced colitis model)	Animal model study (DSS-induced colitis)	[154]
*De Simone Formulation (DSF)*	Effective in UC remission, pouchitis prevention, and post-op CD recurrence	Multiple clinical trials in UC, pouchitis, and CD	[156,157,158,159,160]
*L. acidophilus + L. plantarum + B. lactis + B. breve*	↑ intestinal mucus and goblet cells	Mouse model data	[158]
*L. plantarum + L. acidophilus + L. rhamnosus + E. faecium*	Promotes wound healing and strengthens tight junctions	Experimental study in epithelial wound healing	[159]
*L. salivarius + L. acidophilus + B. bifidum*	↓ recovery time, ↓ disease activity, and improved endoscopic images in UC	Clinical study in UC patients	[160]
*B. infantis + L. acidophilus + E. faecalis ± Bacillus cereus*	Restores gut microbiota balance (mouse model of colitis)	Preclinical study (chronic DSS colitis)	[161]
*Bifidobacterium +* Mesalazine	Ameliorates IBD symptoms, and ↓ adverse effects of mesalazine	Clinical suggestion of synergy with standard treatment	[161]

### 4.4. Next-Generation Probiotics (NGPs)

NGPs represent a pioneering approach to modulating the gut microbiota and managing IBD [162]. Diverging from traditional probiotics, NGPs are sourced from human gut commensals or genetically engineered strains with enhanced functionalities, presenting potential advantages in terms of efficacy and specificity [162]. By leveraging the therapeutic potential of specific microbial strains or engineered organisms, NGPs offer a targeted and personalized approach to treating IBD [162].

One exemplar of an NGP is *F. prausnitzii*, a butyrate-producing bacterium renowned for its potent anti-inflammatory properties [115]. Multiple studies have underscored the therapeutic promise of *F. prausnitzii* in UC, demonstrating reductions in disease activity and inflammation [115].

Another NGP candidate is *Akkermansia muciniphila*, a bacterium specializing in mucin degradation and associated with bolstering gut barrier function and immune regulation [116]. Preclinical investigations have showcased the potential of *A. muciniphila* supplementation in ameliorating colitis and enhancing intestinal barrier integrity in animal models of IBD [116].

*C. butyricum MIYAIRI*, another NGP, is a butyrate-producing bacterium that has shown effectiveness in preventing pouchitis and mitigating alterations in the microbiota of UC patients [117]. Recent findings by Ma et al. indicate that *C. butyricum MIYAIRI-II* could alleviate parameters associated with colitis in a mouse model of DSS-induced colitis [117].

Additionally, genetically engineered probiotic strains offer innovative avenues for IBD therapy [170]. For instance, researchers have developed engineered strains of *Escherichia coli Nissle 1917* that overexpress anti-inflammatory proteins or enzymes involved in metabolite production, such as catalase and superoxide dismutase [171]. These modified strains have exhibited efficacy in reducing inflammation and fostering mucosal healing in preclinical models of IBD [171]. Genetically engineered *E. coli Nissle 1917* has also been shown to enhance the abundance of microbes crucial for maintaining intestinal homeostasis, such as *Lachnospiraceae* and *Odoribacter* [171].

Other NGPs currently under investigation encompass genetically modified *Lactobacilli* and *Bifidobacteria* engineered to produce anti-inflammatory cytokines or metabolites [170,172,173]. These tailored probiotics hold promise for modulating immune responses and reinstating gut homeostasis in individuals with IBD.

In conclusion, NGPs herald a promising frontier in IBD therapeutics, offering targeted interventions that address the underlying mechanisms of disease pathology. Further research and clinical trials are imperative to elucidate the safety, efficacy, and optimal dosing regimens of NGPs in the management of IBD.

### 4.5. Synbiotics

Synbiotics, a combination of probiotics and prebiotics, have garnered interest as a therapeutic approach for IBD [174]. By combining beneficial microorganisms with substrates that promote their growth and activity, synbiotics aim to synergistically enhance the efficacy of the treatment [174].

Several studies have investigated the use of synbiotics in IBD, with promising results [160]. For example, a synbiotic combination of *Lactobacillus* and FOS has been shown to improve clinical outcomes and reduce disease activity in patients with UC [175]. Similarly, another synbiotic formulation containing *Bifidobacterium* and inulin resulted in significant reductions in inflammation and improvements in symptoms in individuals with CD [111].

Furthermore, synbiotics have been found to enhance the efficacy of conventional treatments for IBD [112]. For instance, combining synbiotics with mesalazine, a commonly used medication for IBD, has been shown to improve clinical response rates and reduce the risk of relapse in patients with UC.

Overall, synbiotics represent a promising adjunctive therapy for IBD, offering a multifaceted approach to address the complex interplay between gut microbiota dysbiosis, immune dysfunction, and intestinal inflammation. Further research is needed to optimize synbiotic formulations, dosing regimens, and treatment durations to maximize their therapeutic benefits in IBD management.

### 4.6. Fecal Microbial Transplant (FMT)

FMT is a direct method for reshaping the intestinal microbiota by introducing a fecal suspension from a carefully chosen healthy donor into the intestines of a patient suffering from a disease [113]. Recipients of FMT may inherit crucial genes from the donor that contribute to restoring a healthy and functional gut ecosystem by enhancing the production of SCFAs and restoring immune dysregulation [114]. FMT is already approved in treating recurrent *Clostridioides difficile (C. difficile)* infections (CDI) resistant to antibiotic treatment [176]. While the microbial foundation of IBD proves to be considerably intricate and variable compared to relapsed/refractory CDI, therapies based on the microbiota represent a critical area of exploration for these chronic and incapacitating conditions [177]. Consequently, there has been a surge in clinical studies investigating the effectiveness of FMT in treating IBD [177].

Research has demonstrated the efficacy of FMT in initiating remission among individuals with ulcerative colitis UC [175]. A notable increase in *Bacteroides*, *Proteus*, and *Prevotella* alongside a reduction in *Klebsiella* and *Streptococcus* populations following FMT was discovered by Tian et al. [111].

In a meta-analysis conducted by Colman and Rubin, it was revealed that the remission rate among patients with IBD who underwent FMT was 36.2% [112]. Additionally, they observed a higher remission rate in patients with CD compared to those with UC. Another meta-analysis on FMT for IBD conducted by Paramsothy et al. demonstrated a clinical remission rate of 50.5% [113]. Furthermore, a separate meta-analysis by Caldeira et al. indicated that FMT resulted in a complete remission rate of 37% among IBD patients [114].

Several investigations have been undertaken to evaluate the effectiveness of FMT in initiating remission in UC. For example, Moayyedi et al. conducted a trial involving 75 individuals diagnosed with mild-to-severe UC [176]. The experimental cohort received FMT via enemas from donors, while the control cohort underwent a placebo intervention. Results from the study indicated that patients who underwent FMT achieved clinical remission compared to those in the control group, with statistically significant findings (*p* = 0.03). Another randomized placebo-controlled study, conducted by Paramsothy et al., involved 81 patients with mild-to-moderate UC, with 41 patients in the study group and 40 in the control group [178]. The results indicated a significantly higher rate of endoscopic remission in the study group compared to the control group at week 8 (*p* = 0.021). In the same way, Costello et al. documented a notably superior treatment effect in the study group, consisting of 38 patients with moderate UC who underwent FMT, in contrast to the control group with 35 patients in the placebo arm [179]. Following a two-month follow-up, 12 patients (32%) in the FMT group achieved both clinical and endoscopic remission, whereas only 3 out of 35 patients in the placebo group attained complete remission (*p* = 0.03). Similar results were obtained by Cui et al. showing that FMT improved clinical outcomes in 57% of patients with steroid-dependent UC [180]. These findings were further confirmed by Kunde et al. which found a significant improvement in nine children with UC who received FMT via enema [181].

The efficacy and safety of FMT was also investigated in CD. A pilot single-center trial evaluated the effect of multiple FMTs on 25 CD patients complicated with an intra-abdominal inflammatory mass [182]. All patients received the initial FMT followed by repeated FMTs every 3 months. Clinical response and clinical remission at 3 months post the initial FMT were achieved in 68.0% (17/25) and 52.0% (13/25) of patients, respectively. The percentage of individuals achieving sustained clinical remission with successive FMTs at 6 months, 12 months, and 18 months was 48.0% (12 out of 25), 32.0% (8 out of 25), and 22.7% (5 out of 22), respectively. Radiological healing was attained by 9.5% (2 out of 21) of patients, while 71.4% (15 out of 21) experienced radiological improvement.

Another randomized controlled trial determined the efficacy and safety of different methods of FMT as a potential therapy for CD [183]. A total of 27 patients with CD were randomized to receive FMT by gastroscopy or colonoscopy; a second transplantation was performed 1 week later. Clinical remission, assessed 8 weeks after FMT, was achieved in 18 (66.7%); no significant difference was seen between the two methods. Moreover, microbiota diversity analyses showed that, compared to donors, CD patients showed a significant increase in operational taxonomic units (OUT, 117 vs. 258, *p* < 0.05) 2 weeks after FMT. In CD patients, FMT contributed to increase species richness, raising levels of *Clostridium, Cronobacter, Fusobacterium,* and *Streptococcus*.

Finally, a randomized, single-blind, sham-controlled pilot trial of FMT in adults with colonic or ileo-colonic CD was performed [184]. Out of the enrolled patients, eight underwent FMT, while nine received sham transplantation. The steroid-free clinical remission rates at 10 and 24 weeks post-FMT were 44.4% (4/9) and 33.3% (3/9) in the sham transplantation group, respectively, and 87.5% (7/8) and 50.0% (4/8) in the FMT group. The Crohn’s Disease Endoscopic Index of Severity (CDEIS) exhibited a decrease six weeks after FMT (*p* = 0.03) but not after sham transplantation (*p* = 0.8). Conversely, the C-reactive protein (CRP) level increased six weeks after sham transplantation (*p* = 0.008) but not after FMT (*p* = 0.5). Higher colonization by donor microbiota correlated with the maintenance of remission.

The efficacy of FMT in CD remains less established compared to UC, with mixed results reported in various trials.

Long-term follow-up studies have assessed the durability of FMT-induced remission in IBD patients [185,186]. While some patients maintain remission following FMT, others may experience disease recurrence over time, highlighting the need for further research into optimal maintenance strategies [187].

Nonetheless, diverse clinical investigations aimed at assessing the impact of FMT on IBD have yielded conflicting outcomes, casting uncertainty on its efficacy. Existing data indicate that the effectiveness of FMT in managing IBD cannot be reliably predicted. After 12 weeks of FMT, only one UC patient showed some improvement, as reported by Angelberger et al. [188]. Likewise, Suskind et al. observed no notable improvement in four children who underwent a single FMT administered via a nasogastric tube [189]. The variations in outcomes across clinical trials may stem from differences in disease pathology, donor selection criteria, FMT protocols, and individual responses to treatment. Concerns regarding the safety and effectiveness of FMT have hindered its widespread adoption for IBD management. FMT carries inherent risks, including the potential transmission of infectious agents from the donor to recipient. Instances of severe infections, such as bacteremia and sepsis, have been documented post-FMT, underscoring the necessity for stringent donor screening and safety measures [190]. Whilst most adverse events linked to FMT are mild and transient, such as gastrointestinal discomforts like bloating, diarrhea, and abdominal pain, more severe complications can arise, especially among immunocompromised individuals or those with underlying health conditions.

FMT holds promise as a potential therapeutic intervention for IBD, particularly in UC where it has demonstrated efficacy in inducing remission in some patients. However, further research is needed to elucidate optimal protocols regarding donor selection, administration route, dosing, and long-term maintenance strategies. Additionally, safety considerations and potential adverse events must be carefully evaluated to ensure the overall benefit–risk profile of FMT in IBD patients.

### 4.7. Fecal Virome Transplant (FVT)

In contrast to conventional FMT, FVT entails transferring only gut viruses from healthy donors to diseased patients [191]. Most FVT investigations have been conducted using in vitro mouse models of diseases lacking clear biomarkers, such as obesity and antibiotic-induced dysbiosis [191,192]. Scientists are investigating whether FVT could be an effective treatment for IBD [193]. The rationale behind this approach lies in the idea that viruses in the gut microbiota, particularly bacteriophages, notably impact overall bacteriome compositions, altering *Firmicutes–Bacteroidetes* ratios, diversity, and specific bacterial abundances, albeit the latter contributing minimally to the bacteriome.

Research on FVT in inflammatory bowel disease is still in its early stages, but there is growing interest in exploring its potential therapeutic applications. FVT offers advantages over FMT by reducing the risk of transferring unknown pathogens or bacteria with undesirable functionalities.

In a study by Ott et al., FVT preparations, which underwent sterile filtration, were administered to five patients with CDI, including three who had failed FMT and/or antibiotic treatments and one deemed ineligible for FMT due to infectious risks [194]. All five patients recovered from CDI infection post-FVT and remained symptom-free for at least 6 months. Although virome analysis was conducted on only one patient, significant changes were observed in the patient’s phageome, resembling that of the donor. Nonetheless, due to the study’s focus on the efficacy of fecal filtrates rather than the virome specifically, and the limited sample size, no definitive causal links between the virome and patient recovery could be established, nor were specific beneficial phages identified. Therefore, while the therapeutic potential of the virome has been demonstrated, progress in developing virome-based therapies is contingent upon a better understanding of the taxa and mechanisms by which viruses impact host metabolism, influencing both diseased and healthy gut states.

Safety is a critical consideration in FVT, as with any transplantation procedure. There are concerns about the potential transfer of harmful viruses or genetic elements from donors to recipients. Therefore, stringent donor screening protocols and safety measures are essential to minimize the risk of adverse effects. Additionally, researchers are exploring methods to isolate and characterize specific beneficial phages that could be used in targeted therapies, thereby reducing the need for whole-virome transplants.

### 4.8. Phage Therapy

Bacteriophages, or phages, have garnered attention as a potential alternative to antibiotics for combating multidrug-resistant bacteria [195]. In the realm of gastrointestinal disorders, phage therapy, which involves administering cocktails of bacteriophages, shows promise for conditions associated with specific bacterial colonization or infection, such as IBD with *AIEC*, *Klebsiella pneumoniae*, and *C. difficile* [196,197].

Compared to fecal transplants or antibiotics, phage therapies offer advantages by enabling the targeting of specific commensal bacteria, including drug-resistant strains [198]. This targeted approach helps to minimize unintended alterations in the gut microbiota without the transfer of live bacteria.

In experimental murine models of IBD deliberately colonized with pathobionts linked to IBD, researchers have examined diverse combinations of bacteriophages. Although these studies have typically demonstrated efficacy in eliminating targeted bacteria, assessing their clinical implications on disease activity beyond infection resolution presents challenges [199]. Moreover, the limitations of animal models hinder the direct extrapolation of these outcomes to human cases of IBD. Additionally, numerous investigations into phage therapy for IBD have concentrated on targeting individual bacterial strains, thereby neglecting the complex microbial diversity characteristic of IBD.

### 4.9. Targeting Archaeome

Targeting the archaeome presents significant challenges as human methanogenic archaea have demonstrated high resistance to antibiotics, rendering them susceptible only to compounds that also affect bacteria and eukarya [200]. This limitation complicates the development of specific therapies. However, recent discoveries have indicated that statins can inhibit archaeal cell membrane biosynthesis without affecting bacterial populations, suggesting a potential avenue for targeted therapeutic intervention while preserving the integrity of the intestinal microbiota. This finding offers a potential starting point for modulating the archaeome in patients with IBD. However, achieving success in this endeavor will require extensive further research and dedicated efforts to refine IBD treatment strategies.

### 4.10. Targeting Microbiome

When it comes to modifying fungal composition, interventions may entail the administration of specific antifungal medications. For example, a recent small-scale pilot study, NCT03476317, has completed patient enrollment to evaluate the effects of a novel therapeutic regimen targeting the gut microbiota. This regimen involves bowel lavage and antibiotic treatment, with or without the inclusion of the antifungal drug fluconazole. The study aims to evaluate the efficacy of this approach in treating active CD or indeterminate colitis (IBDU) that has shown resistance to conventional immunosuppressive therapy.

In forthcoming clinical trials such as NCT05049525, which is currently not enrolling participants, the objective is to evaluate the effectiveness of combined antifungal therapies, including itraconazole and terbinafine, compared to a placebo in patients diagnosed with CD. This trial aims to furnish additional evidence supporting the idea that targeting fungal components in these patients may contribute to achieving remission. Similarly, the pilot study NCT04966585, also not recruiting yet, aims to investigate whether the changes in microbial composition induced by antifungal treatment are associated with reduced downstream immune responses in CD patients who possess a genetic predisposition to mounting robust immune reactions against *Malassezia*.

In the realm of fungal-derived factors, a randomized clinical trial examining the efficacy of *Saccharomyces boulardii* conducted by Garcia Vilela et al. revealed heightened disease activity index scores among a cohort of CD patients [200]. Following studies in CD patients have similarly indicated enhancements in relapse rates and intestinal permeability.

## 5. Conclusions

In summary, the interaction between the gut microbiota and the immune system plays a pivotal role in the pathophysiology of IBD. The stability of the gut microbial structure is crucial for numerous physiological processes, including direct interaction with the intestinal epithelium and modulation of mucus secretion and mucosal immunity. Understanding these intricate dynamics and the specific role of each microbiota component implicated in IBD is essential for elucidating disease mechanisms and developing innovative therapeutic strategies.

Manipulating the microbiota–immune axis holds promise for more effective IBD management and improved patient outcomes, spanning from preventive measures to novel treatments. Changes in the gut microbiota structure and metabolites are emerging as key parameters for drug development and mechanistic studies. Restoring the gut microbiota structure can alleviate IBD exacerbations, with potential interventions including the colonization of beneficial bacteria or the supplementation of commensal-produced metabolites to enhance intestinal barrier integrity in IBD patients. Advanced metagenomic and metabolomic analyses provide profound insights into the complex relationships among gut microbes, metabolites, and hosts.

Continued research efforts are necessary to explore nonpharmacological and behavioral interventions aimed at restoring gut ecosystem homeostasis and immune function, ultimately offering relief to individuals with IBD. Additionally, the standardization of research methods for studying gut microbiota is imperative to ensure reproducible data and robust knowledge. Establishing guidelines and achieving consensus on research protocols will facilitate the generation of reliable data. Furthermore, large-sample-size cohorts are crucial to address the inherent variability in microbiota-related research, necessitating patient stratification based on geographical origin, sex, clinical characteristics, and lifestyle habits to mitigate study inconsistencies.

In this context, future investigations should prioritize a deeper functional understanding of host–microbiota interactions through integrated multi-omics platforms, including metagenomics, metabolomics, and transcriptomics, to decipher the mechanisms by which specific microbes and their metabolites influence intestinal immunity. Equally important is the identification of microbial signatures capable of predicting therapeutic response, which may guide the development of tailored interventions. To fully realize the clinical potential of microbiota-targeted therapies, standardized protocols for interventions such as fecal microbiota transplantation and next-generation probiotics—covering aspects like donor selection, dosage, administration routes, and follow-up—must be implemented. Additionally, exploring synergistic combinations of microbial and immunomodulatory therapies could enhance therapeutic outcomes, particularly in early or refractory disease stages. Ultimately, well-designed randomized controlled trials with clearly defined clinical and molecular endpoints are essential to validate the efficacy and safety of these approaches and to integrate them into personalized treatment strategies for IBD.

The following crucial unanswered questions remain: Can specific microbial metabolites serve as predictive biomarkers for therapeutic response in IBD? And to what extent can targeted microbial manipulation reshape the immune landscape in a durable and disease-specific manner? Addressing these questions will be key to translating microbiome science into clinically actionable strategies.

## Figures and Tables

**Table 1:** Microbiota-directed interventions in IBD.

Intervention	Proposed Mechanism of Action	Clinical Evidence/Applications	Key Limitations	References
Probiotics	Microbial balance restoration; barrier enhancement; and immune modulation via cytokines and SCFAs.	Moderate evidence in UC; limited efficacy in CD; and some strains used with mesalamine.	Strain-specific effects; lack of standard dosing; variable formulations; and modest impact on CD.	[109,110]
Fecal microbiota transplantation (FMT)	Broad microbial reconstitution; suppression of pathobionts; and modulation of T-cells and metabolism.	Positive results in UC; and under investigation in CD and pouchitis.	Donor/protocol variability; regulatory issues; and unclear long-term safety.	[111,112,113,114]
Next-generation probiotics (NGPs)	Targeted strains with immune/metabolic functions.	Promising preclinical and early trial data (e.g., *F. prausnitzii*, *A. muciniphila*).	Limited availability; stability issues; and regulatory uncertainty.	[115,116,117]
Dietary interventions (e.g., CDED)	Exclusion of pro-inflammatory foods; and promotion of beneficial taxa.	Effective in pediatric CD; good adherence; and microbiome/metabolome shifts observed.	Limited adult data; long-term adherence challenging; and no universal guidelines.	[118,119,120,121]

## Data Availability

Not applicable.

## Abbreviations

AID	Anti-Inflammatory Diet
AhR	Aryl hydrocarbon receptor
AMPs	Antimicrobial peptides
ASBT	Apical sodium-dependent bile acid transporter
ATG	Autophagy-related gene
BAs	Bile acids
BSH	Bile salt hydrolase
CARD	Caspase recruitment domain
CD	Crohn’s disease
CDED	Crohn’s Disease Exclusion Diet
CRC	Colorectal cancer
DCs	Dendritic cells
DSF	De Simone formulation
EEN	Exclusive enteral nutrition
ECCO	European Crohn’s and Colitis Organization
ETBF	Enterotoxigenic *Bacteroides fragilis*
FMT	Fecal microbiota transplantation
FODMAP	Fermentable oligo-, di-, monosaccharides and polyols
FVT	Fecal virome transplantation
GOSs	Galactooligosaccharides
GPR	G protein-coupled receptor
IBD	Inflammatory bowel disease
IBD-AID	IBD-specific Anti-Inflammatory Diet
IBDU	IBD unclassified
IECs	Intestinal epithelial cells
IgA	Immunoglobulin A
IL	Interleukin
NGPs	Next-generation probiotics
NLRs	NOD-like receptors
NOD	Nucleotide-binding oligomerization domain
PAMPs	Pathogen-associated molecular patterns
PEN	Partial enteral nutrition
PRRs	Pattern recognition receptors
PXR	Pregnane X receptor
ROS	Reactive oxygen species
SBAs	Secondary bile acids
SCFAs	Short-chain fatty acids
SCD	Specific carbohydrate diet
SRB	Sulfate-reducing bacteria
TCA	Tricarboxylic acid
TGF-β	Transforming growth factor beta
Th	T helper
TLRs	Toll-like receptors
TNF-α	Tumor necrosis factor alpha
TJs	Tight junctions
Tregs	Regulatory T-cells
UC	Ulcerative colitis
VDR	Vitamin D receptor
VLPs	Viral-like particles
